# Enriching inventories of rare genera: five new species of Pteromalidae (Hymenoptera, Chalcidoidea) from Cyprus

**DOI:** 10.3897/zookeys.1286.196157

**Published:** 2026-07-23

**Authors:** Evangelos Koutsoukos, Mircea-Dan Mitroiu

**Affiliations:** 1 Laboratory of Vector Ecology and Applied Entomology, Joint Services Health Unit Cyprus, BFC RAF Akrotiri BFPO 57, Akrotiri, Cyprus Faculty of Biology, Alexandru Ioan Cuza University Iasi Romania; 2 Enalia Physis Environmental Research Centre, Eleftherias 1, Xylotymvou, 7510, Larnaca, Cyprus Section of Zoology and Marine Biology, Department of Biology, National and Kapodistrian University of Athens Athens Greece; 3 Section of Zoology and Marine Biology, Department of Biology, National and Kapodistrian University of Athens, 15784 Athens, Greece Zoology Museum, Department of Biology, National and Kapodistrian University of Athens Athens Greece; 4 Zoology Museum, Department of Biology, National and Kapodistrian University of Athens, Athens, Greece Laboratory of Vector Ecology and Applied Entomology, Joint Services Health Unit Cyprus Akrotiri Cyprus; 5 Faculty of Biology, Alexandru Ioan Cuza University, Iasi, Romania Enalia Physis Environmental Research Centre Larnaca Cyprus

**Keywords:** *
Ablaxia
*, *
Erythromalus
*, *
Hemitrichus
*, *
Janssoniella
*, parasitoid

## Abstract

Four new genera of the family Pteromalidae (*Ablaxia* Delucchi, 1957, *Erythromalus* Graham, 1956, *Hemitrichus* Thomson, 1878, and *Janssoniella* Kerrich, 1957) are reported for the fauna of Cyprus, and five new species are described: *Ablaxia makrisi* Koutsoukos & Mitroiu, **sp. nov**., *Ablaxia toxeftrae* Koutsoukos & Mitroiu, **sp. nov**., *Erythromalus makrisi* Koutsoukos & Mitroiu, **sp. nov**., *Hemitrichus akrotiriensis* Koutsoukos & Mitroiu, **sp. nov**., and *Janssoniella aphrodite* Koutsoukos & Mitroiu, **sp. nov**. While the ecology of all the new species is unknown, we provide and illustrate the diagnostic characters that separate the new species from the existing ones to assist in their future identification. The discovery of these new species highlights the knowledge shortfalls for many chalcid wasp groups in Southeastern Europe and the Middle East.

## Introduction

Until recently, the family Pteromalidae contained 33 subfamilies and more than 640 genera, serving as a polyphyletic “garbage bin” of taxa that did not fit anywhere else within Chalcidoidea (Burks et al. 2022). Still, Pteromalidae (*sensu stricto*) contains more than 420 genera and 3000 described species and is one of the largest and diverse families within Chalcidoidea (Burks et al. 2025). While there have been various studies regarding the Palaearctic Pteromalidae, such as the monography of Graham (1969), most genera remain poorly known, and more and more species and genera have been described the last decades (Bouček and Rasplus 1991; Tselikh et al. 2025a, 2025b). This is quite evident for many regions of the Southeastern Europe and the Middle East, where the collecting effort and studies regarding chalcid wasps have been more scarce compared to Western Europe.

The genus *Ablaxia* Delucchi, 1957 includes one Nearctic, two Neotropical and six Western Palaearctic (European) species (UCD community 2023); all but one of the six European species are included in the revision of Graham (1969); however, the description of these two species, as well as the detection of other undescribed species from the area (Koutsoukos and Mitroiu unpublished data), indicate that the genus is severely understudied and needs a revision. *Erythromalus* Graham, 1956 has only two described species with a wide distribution in Europe, but their ecology is largely unknown (Graham 1969; Haas et al. 2021; UCD Community 2023). *Hemitrichus* Thomson, 1878 is a genus with a unique combination of morphological characters, which makes its assignment to one of the existing subfamilies difficult. It includes five species (one Nearctic, one from the Russian Far East, one from the Middle East, and two European) (Tselikh 2015; UCD Community 2023). Finally, *Janssoniella* Kerrich, 1957 was revised by Tselikh (2020), who included a key with six species present in the Eastern Palaearctic; *J. ambigua* Graham, 1969, *J. caudata* Kerrich, 1957 and *J. major* Kerrich, 1957 were keyed by Graham (1969), while *J. intermedia* Hedqvist, 1968 is not present in either key.

Cyprus is the third largest island of the Mediterranean, situated between three continents (Africa, Asia, and Europe). While it covers a small area of 9,251 km^2^, its topography and climate contribute to its high endemism and habitat richness. Estimations on its insect biodiversity reach more than 6,000 species, with many being endemic (Department of Forests 2023). However, Cyprus remains largely understudied, especially regarding chalcid wasps. Nevertheless, there have been recent advances on the study of other hymenopteran groups such as ants and bees (Varnava et al. 2020; Rosa and Makris 2023; Salata et al. 2023a, 2023b, 2024; Koutsoukos et al. 2024). The Universal Chalcidoidea Database lists only 11 species of the family Pteromalidae from Cyprus, which is a severe underestimation of its true fauna (UCD community 2023). In the framework of a two-year Darwin-Plus fellowship funded by the British Government, we aimed on filling in the gaps of this biodiversity knowledge shortage by collecting within Akrotiri U.K. Sovereign Base Area (SBA) and other regions of Cyprus. Here, we describe five new species belonging to four rarely collected genera (*Ablaxia*, *Erythromalus*, *Hemitrichus*, and *Janssoniella*) of the family Pteromalidae.

## Methods

Examined specimens were collected from various localities in Cyprus (provinces Limassol, Nicosia, and Paphos), and U.K. SBA of Akrotiri between 2024 and 2025. Specimens were collected both in natural and urbanized habitats. Collections were conducted either by sweeping, by hand, or beating-sheet with the use of an entomological umbrella. Following Heraty and Hawks (1998), specimens were dried with the use of hexamethyldisilazane (HMDS) and card-mounted for examination. Classification follows Burks et al. (2022), and terminology follows Graham (1969) and Burks et al. (2025). The antennal formula includes the terminal button (the 12^th^ flagellomere). The mandibular formula indicates the number of teeth in the left and right mandibles. Observations were made using a Leica S8APO stereomicroscope. Images were taken using a Leica DFC500 digital camera attached to a Leica M205A automated research stereomicroscope, or a Nikon Z5 camera with a Laowa 25 mm f/2.8 2.5–5 × ultra macro on Cognisys Stackshot Macro rail, assembled with Zerene Stacker®, and further processed with Adobe® Photoshop® v. 7.0 to enhance their clarity.

Abbreviations: **F** = funicular segment; **LOL** = lower ocular line; **M** = marginal vein; **OOL** = shortest distance between posterior ocellus and eye margin, dorsal view; **P** = postmarginal vein; **POL** = shortest distance between posterior ocelli, dorsal view; **S** = stigmal vein. The height of the stigma was measured on a latitudinal axis starting from its lowest point, reaching its upper margin, and its distance from the P was measured from this upper point to the lowest point of P, on a latitudinal axis. Specimens will be deposited in the Museum of Zoology at the National and Kapodistrian University of Athens (**ZMUA**), and Mitroiu personal collection (**MICO**).

## Results

**Subfamily Pteromalinae**

**Genus *Ablaxia* Delucchi, 1957**

### Ablaxia makrisi

Taxon classificationAnimaliaHymenopteraPteromalidae

Koutsoukos & Mitroiu sp. nov.

B7738B14-37D6-559F-8F66-B00FC41465BC

https://zoobank.org/43263468-82A9-4D89-B508-86D3F74DF1A0

Fig. 1

#### Material examined.

Holotype female: Cyprus • Paphos, Agios Nikolaos, Gefiri tou Tzelefou, 34.8892, 32.7473, 15.xi.2025, leg. Makris Christodoulos, “ZMUA000316739” (ZMUA).

**Figure 1.**
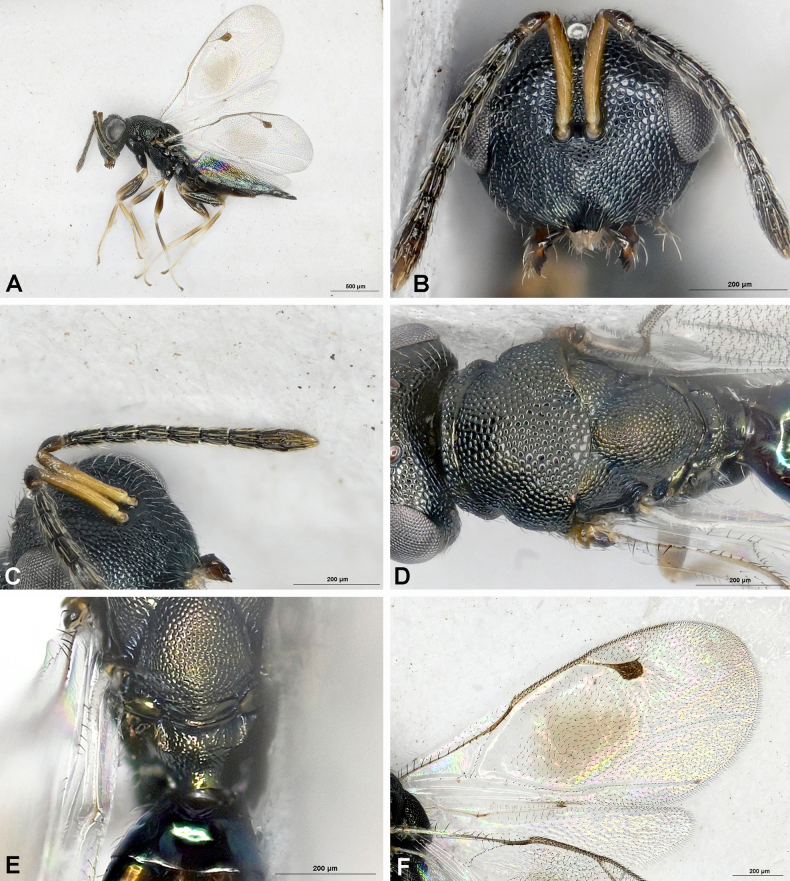
*Ablaxia makrisi* sp. nov. holotype ♀. **A**. Habitus; **B**. Head, frontal view; **C**. Left antenna, anterolateral view; **D**. Mesosoma, dorsal view; **E**. Scutellum, propodeum, dorsal view; **F**. Fore wing.

#### Diagnosis.

POL 1.41 × OOL; temples slightly more than 0.2 × eye length; pedicel length 0,81 × F1 length; F1 2.7 × as long as broad, F6 1.6 × as long as broad; mesoscutum 1.38 × as wide as long; scutellum with coarse reticulation; gaster 3.09 × as long as broad.

#### Description.

**Female**. Body length 2.4 mm.

Colour. Head and mesosoma black with dark blue and dark green metallic reflections; scutellum and axillae predominantly dark green, with traces of bronze reflections; metasoma including ovipositor sheath dark brown, dorsal side of tergites with bluish reflections; antenna with scape gradually changing from testaceous to fuscous from base to tip; pedicel and flagellum dark brown; all coxae as the lateral side of mesosoma, all femora dark brown, with slight metallic reflections (more evidently on hind femora); fore tibia testaceous ventrally to infuscate dorsally, mid tibia dark brown on outer side, testaceous on inner side, except for white tips; hind tibia with infuscate band mostly in middle part, remainder white; fore tarsus infuscate with 5^th^ tarsomere brownish, mid and hind tarsi pale yellow, with brownish tip; fore wing with a pale brownish discal cloud below marginal vein, venation greyish, setae dark brown; setae white on head, light brown on mesosoma.

Head in dorsal view 2.35 × as broad as long and 1.32 × as broad as mesoscutum; in frontal view 1.30 × as broad as high. In dorsal view temple 0.21 × as long as eye. POL 1.41 × OOL. Eye height 1.24 × eye length and 1.61 × as long as malar space. Oral fossa 0.41 × as long as head width. Clypeus radially striate, its lower margin slightly emarginate. Mandibular formula 4:4. Antenna 11264, inserted above LOL; scape nearly as long (0.96 ×) as eye height, reaching level of vertex; pedicel 0.81 × as long as F1; combined length of pedicel and flagellum 1.16 × breadth of head; flagellum almost filiform, all funiculars longer than broad; F1 2.7 × as long as broad, F6 1.6 × as long as broad; clava 2.63 × as long as broad.

Mesosoma 1.67 × as long as broad and 1.62 × as long as high. Mesoscutum 1.38 × as broad as long. Mesoscutellum 1.18 × as long as broad, with coarse and dense sculpture. Propodeum medially 0.56 × as long as mesoscutellum; median carina meeting costula, both fine; basal foveae present; plicae absent; median area raised along costula, densely reticulate; nucha well developed, reticulate. Fore wing 2.18 × as long as broad; basal fold with complete row of setae; basal cell bare; speculum open below; M 0.93 × as long as P and 1.44 × as long as S; stigma large, 0.72 × the distance to P, uncus short.

Gaster lanceolate, longer than head plus mesosoma, 3.09 × as long as broad. Hypopygium in proximal third of gaster. Ovipositor sheath projecting slightly beyond apex of metasoma.

**Male**. Unknown.

#### Distribution.

Cyprus.

#### Host.

Unknown.

#### Etymology.

Named after the collector, Mr Christodoulos Makris, Cypriot entomologist (noun in genitive case).

#### Notes.

*Ablaxia makrisi* ends up in the 3^rd^ couplet of Graham (1969), where it does not fit any of the two options, since the gaster is 3.09 × as long as broad, and the temples in dorsal view are slightly more than 1/5 of the eye length. It differs from *A. toxeftrae* sp. nov. by the gaster length: width ratio (2.7 × in *A. toxeftrae*), the POL:OOL ratio, (1,41 × in *A. makrisi*, 1.71 × in *A. toxeftrae*), the pedicel length to F1 length ratio (0,81 × in *A. makrisi*, as long as in *A. toxeftrae*), the length: width ratios of the funicular segments (F1 1.83 × as long as broad, F6 1.11 × as long as broad in *A. toxeftrae*, F1 2.7 × as long as broad, F6 1.6 × as long as broad in *A. makrisi*), the mesoscutum width: length ratio (1.38 × in *A. makrisi*, 1.71 × in *A. toxeftrae*), and the sculpture of the scutellum (much coarser reticulation in *A. makrisi*, the cells much smaller than those of *A. toxeftrae*).

### Ablaxia toxeftrae

Taxon classificationAnimaliaHymenopteraPteromalidae

Koutsoukos & Mitroiu sp. nov.

26646D90-2D03-54A8-A7C0-C7A33F74C779

https://zoobank.org/CC2EEA74-E3A4-483D-A5AF-752036A17AD2

Fig. 2

#### Material examined.

Holotype female: Cyprus • Paphos, Akamas, near Toxeftra Beach, 34.918, 32.332, 16.ii.2024, swept on *Smyrnium olusatrum* L., leg. Koutsoukos E., Demetriou J., “ZMUA000316740” (ZMUA).

**Figure 2.**
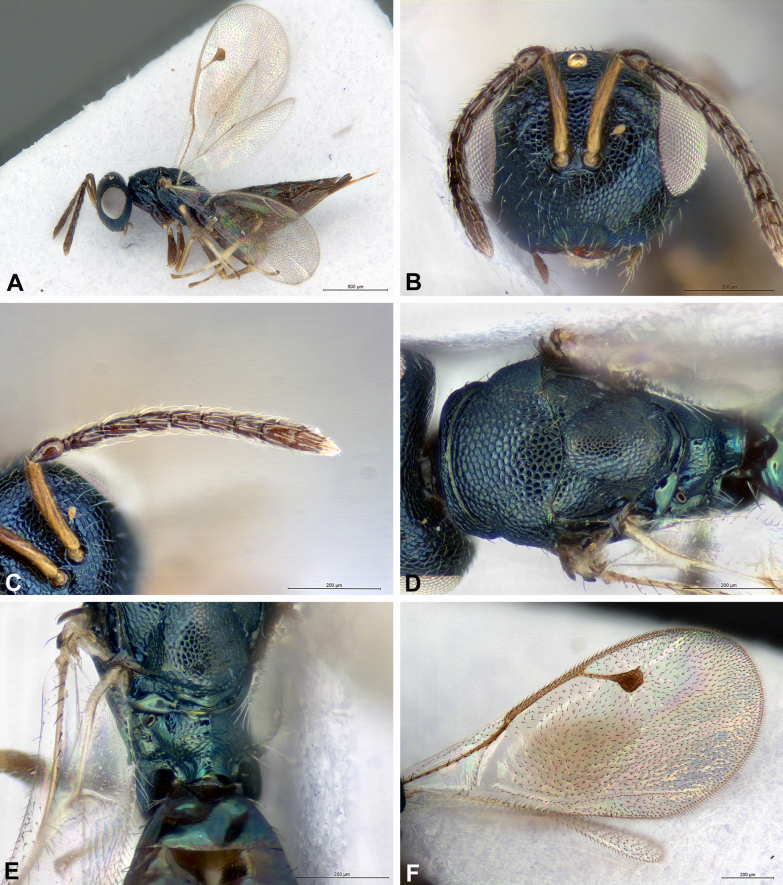
*Ablaxia toxeftrae* sp. nov. holotype ♀. **A**. Habitus; **B**. Head, frontal view; **C**. Left antenna, anterolateral view; **D**. Mesosoma, dorsal view; **E**. Scutellum, propodeum, dorsal view; **F**. Fore wing.

#### Diagnosis.

POL 1.71 × OOL; temples 0.18 × eye length; pedicel length about as long as F1 length; F1 1.83 × as long as broad, F6 1.11 × as long as broad; mesoscutum 1.71 × as wide as long; gaster 2.71 × as long as broad.

#### Description.

**Female**. Body length 1.7 mm.

Colour. Head and mesosoma black with dark-blue metallic reflections; metasoma, including ovipositor sheath, dark brown; dorsal side of tergites with bluish reflections; antenna with scape gradually changing from light brown to brown from base to tip; pedicel and flagellum dark brown; all coxae as mesosoma, all femora dark brown, with slight metallic reflections; fore and mid tibiae mostly brown, hind tibia with a brown band in proximal half, remainder pale yellow; fore tarsus brown, mid and hind tarsi proximally pale yellow, remainder brown; fore wing with a pale-brownish discal cloud, venation and setae dark brown; setae white on head, light brown on mesosoma.

Head in dorsal view 2.19 × as broad as long and 1.27 × as broad as mesoscutum; in frontal view 1.31 × as broad as high. In dorsal view temple 0.18 × as long as eye. POL 1.71 × OOL. Eye height 1.23 × eye length and 1.75 × as long as malar space. Oral fossa 0.47 × as long as head width. Clypeus radially striate, its lower margin emarginate. Antenna 11264, inserted above LOL; scape 0.9 × as long as eye height, reaching level of vertex; pedicel about as long as F1; combined length of pedicel and flagellum 1.23 × breadth of head; flagellum almost filiform, all funiculars longer than broad; F1 1.83 × as long as broad, F6 1.11 × as long as broad; clava 3.33 × as long as broad.

Mesosoma 1.52 × as long as broad and 1.61 × as long as high. Mesoscutum 1.71 × as broad as long. Mesoscutellum as long as broad. Propodeum medially 0.55 × as long as mesoscutellum; median carina meeting costula, both fine; basal foveae present; plicae absent; median area raised along costula, mainly reticulate; nucha well developed, reticulate. Fore wing 2.23 × as long as broad; basal fold with complete row of setae; basal cell bare; speculum open; M 0.9 × as long as P and 1.42 × as long as S; stigma very large, almost as high as distance to P, uncus short.

Gaster lanceolate, longer than head plus mesosoma, 2.71 × as long as broad. Hypopygium in proximal third of gaster. Ovipositor sheath projecting slightly beyond apex of metasoma.

**Male**. Unknown.

#### Distribution.

Cyprus.

#### Host.

Unknown.

#### Etymology.

Named after the locality it was collected near, Toxeftra Beach, near Avakas Gorge in Akamas National Park (noun in genitive case).

#### Notes.

As *Ablaxia makrisi*, *A. toxeftrae* sp. nov. runs to couplet 3 in the key of Grahams (1969), although it is not possible to proceed to step 4 or 5, since the gaster is 2.7 × as long as broad, and the temples are only 0.18 × as the eye. It differs from *A. makrisi* by the POL:OOL ratio (1,41 × in *A. makrisi*, 1.71 × in *A. toxeftrae*), the pedicel length to F1 length ratio (0.81 × in *A. makrisi*, as long as in *A. toxeftrae*), the length/width ratios of the funicular segments (F1 1.83 × as long as broad, F6 1.11 × as long as broad in *A. toxeftrae*, F1 2.7 × as long as broad, F6 1.6 × as long as broad in *A. makrisi*), the mesoscutum width:length (1.38 × in *A. makrisi*, 1.71 × in *A. toxeftrae*), the sculpture of the scutellum (much coarser reticulation in *A. makrisi*, the cells much smaller than those of *A. toxeftrae*), and the gaster length:width ratio (3.09 × in *A. makrisi*, 2.71 × in *A. toxeftrae*).

##### Genus *Erythromalus* Graham, 1956

### Erythromalus makrisi

Taxon classificationAnimaliaHymenopteraPteromalidae

Koutsoukos & Mitroiu sp. nov.

D34348E7-9B0D-56BB-88FC-2C2EA3072195

https://zoobank.org/F47DA0F4-97CE-4884-830E-D9685B77194A

Fig. 3

#### Material examined.

Holotype female: Cyprus • Nicosia, Agios Sozomenos, 35.065, 33.439, 3.xii.2025, leg. Makris, C., “ZMUA000316741” (ZMUA).

**Figure 3.**
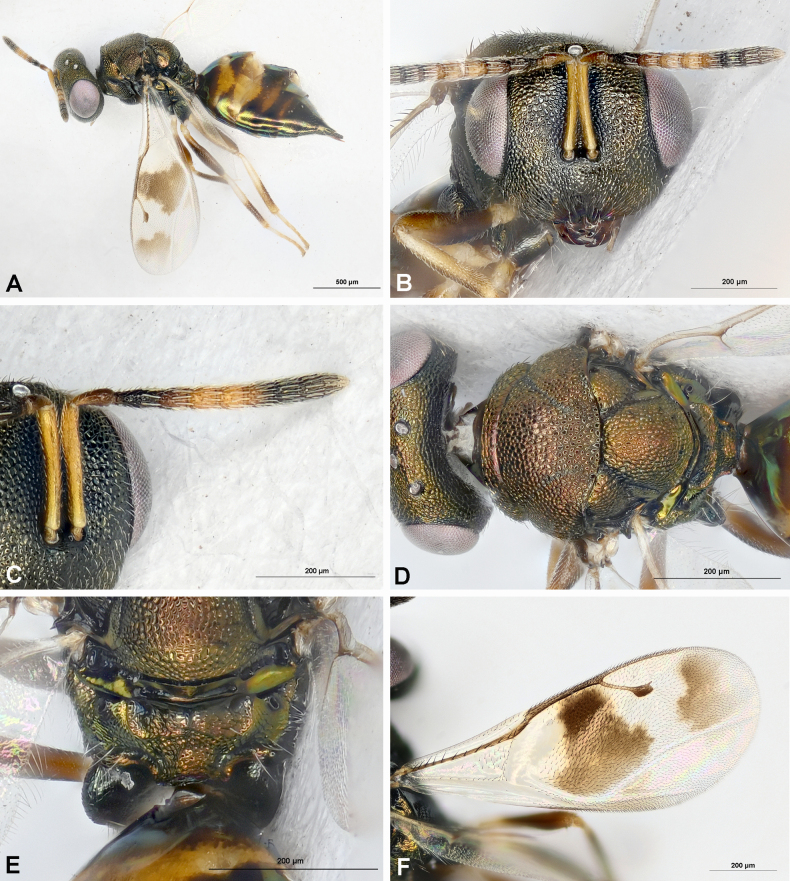
*Erythromalus makrisi* sp. nov. holotype ♀. **A**. Habitus; **B**. Head, frontal view; **C**. Left antenna, anterolateral view, **D**. Mesosoma, dorsal view, **E**. Scutellum, propodeum, dorsal view; **F**. Fore wing.

#### Description.

**Female**. Body length 2.4 mm.

Colour. Head and mesosoma dark green, with bronze metallic reflections; metasoma dark green, with some testaceous bands on dorsal side of tergites 1–4 and with those on tergites 1 and 2 more evident than those on tergites 3 and 4; ovipositor sheaths dark brown, cercal setae pale; antenna with scape gradually changing from light yellow to testaceous from base to tip; pedicel and anelli dark brown; F1 nearly black; F2 dark brown basally, testaceous distally; F3 and F4 testaceous; F5 basally testaceous, distally dark brown; F6 dark brown; clava dark brown, except for its apical part where it tends to become testaceous; all coxae as mesosoma; femora fuscous except for their bases which are whitish yellow; tibiae whitish yellow on basal half, dark brown on distal half, testaceous apically; first tarsomere whitish, remainder light brownish; fore wing with two fuscous clouds, one starting from marginal vein, nearly reaching hind margin of wing; other cloud close to apical margin, covering apical half of wing height; setae white on lower part of head, dark on rest of the body.

Head in dorsal view 2.27 × as broad as long and 1.16 × as broad as mesoscutum; in frontal view 1.26 × as broad as high. In dorsal view temple 0.2 × as long as eye. POL 1.42 × OOL. Eye height 1.39 × eye length and 2.3 × as long as malar space. Oral fossa 0.41 × as long as head width. Clypeus radially striate, its lower margin deeply emarginate. Antenna 11264, inserted above LOL; scape 0.78 × as long as eye height, not reaching median ocellus; pedicel 0.89 × as long as F1; combined length of pedicel and flagellum 0.94 × breadth of head; flagellum almost filiform, all funiculars longer than broad with one row of sensilla; F1 2.16 × as long as broad, F6 1.05 as broad; clava 2.56 × as long as broad.

Mesosoma 1.36 × as long as broad and 1.54 × as long as high. Pronotum 0.19 × long as mesoscutum. Mesoscutum 1.85 × as broad as long. Mesoscutellum as long as broad. Propodeum medially 0.58 × as long as mesoscutellum; median carina present, meeting costula; plicae absent; median area strongly reticulate; nucha developed, reticulate. Fore wing 2.34 × as long as broad; basal fold with complete row of setae; basal cell pilose in its distal third; speculum open; M 0.91 × as long as P and 1.51 × as long as S; stigma moderate, its height 0.5 × as long as distance to P, uncus short.

Gaster ovate, pointed at apex, 1.12 × as long as head plus mesosoma, 1.92 × as long as broad. Last tergite, 1.45 × as long as its basal breath. Hypopygium reaches slightly beyond middle of gaster. Ovipositor sheath projecting very slightly beyond apex of metasoma.

**Male**. Unknown.

#### Distribution.

Cyprus.

#### Host.

Unknown.

#### Etymology.

Named after the collector, Mr Christodoulos Makris, Cypriot entomologist (noun in genitive case).

#### Notes.

*Erythromalus makrisi* sp. nov., demonstrates a combination of characters between the two described species of the genus in the key of Graham (1969). While its combined length of the pedicel and flagellum is shorter that the breadth of the head, which agrees with *Erythromalus rufiventris* (Walker, 1835), the basal cell of fore wing has mostly its distal third pilose, and the gaster is slightly longer than head plus mesosoma. Moreover, it differs from both species by the striking colouration of its antenna, as well as the overall colouration (mesosoma, gaster).

##### Subfamily Trigonoderinae Bouček, 1964

**Genus *Janssoniella* Kerrich, 1957**

### Janssoniella aphrodite

Taxon classificationAnimaliaHymenopteraPteromalidae

Koutsoukos & Mitroiu sp. nov.

38B6EFC9-B7F8-523C-A7D3-DC677E84E4F8

https://zoobank.org/A17F1E31-79A2-458B-9131-03F1A384677C

Fig. 4

#### Material examined.

Holotype female: Cyprus • Paphos, Yeroskipou, 34.763, 32.440, 27.iii.2024, beating-sheet on *Ferula communis* L., leg. Koutsoukos E., Demetriou J., “ZMUA000316742” (ZMUA).

**Figure 4.**
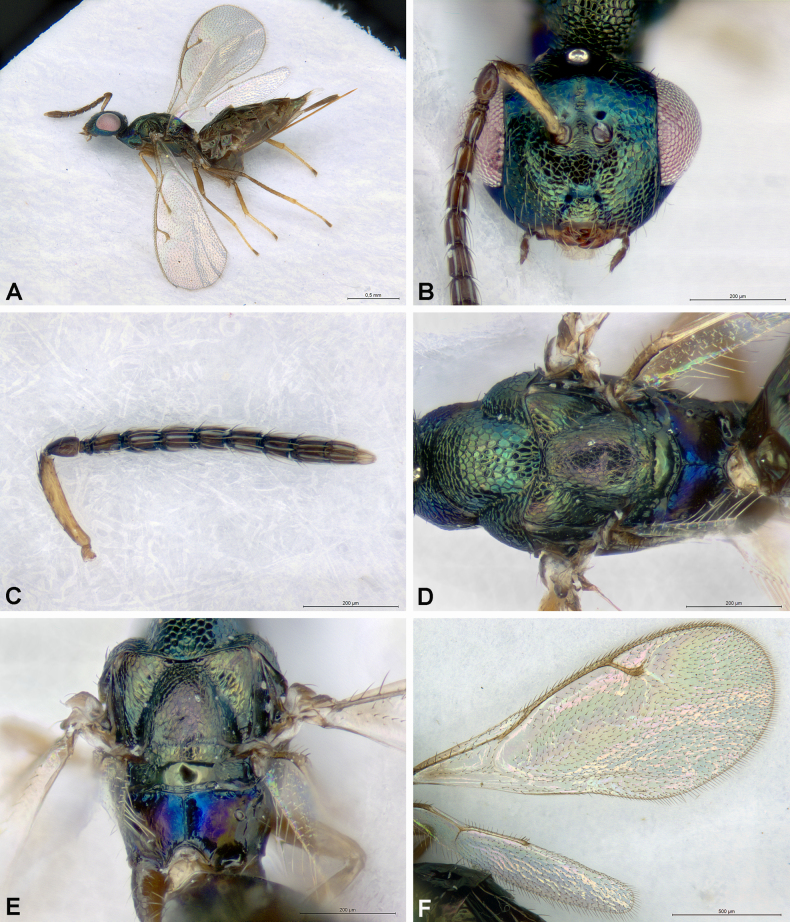
*Janssoniella aphrodite* sp. nov. holotype ♀. **A**. Habitus; **B**. Head, frontal view; **C**. Left antenna, anterolateral view; **D**. Mesosoma, dorsal view; **E**. Scutellum, propodeum, dorsal view; **F**. Fore wing.

#### Description.

**Female**. Body length 2 mm.

Colour. Head and mesosoma mainly metallic green; head with some bluish reflections mainly on upper face; lateral sides of pronotal collar violet-blue; anterior part of axillae and mesoscutellum mainly dark bronze; propodeum bright violet blue; metasoma including ovipositor sheath dark brown, first tergite and syntergum with weak metallic reflections; antenna dark brown except scape mainly pale yellow; all coxae brown, hind coxa with some metallic reflections; remainder of legs brown, except mid and hind tarsi paler and protarsi darker; fore wing hyaline, slightly infumate in proximal part of basal cell; wing venation and setae brown; setae whitish on head, almost absent on mesosoma.

Head in dorsal view 2.26 × as broad as long and 1.34 × as broad as mesoscutum; in frontal view 1.22 × as broad as high. In dorsal view temple 0.26 × as long as eye. POL 1.25 × OOL. Eye height 1.33 × eye length and twice as long as malar space. Oral fossa 0.37 × as long as head width. Tentorial pits deep. Antenna 11264, inserted above LOL. Clypeus from smooth near its anterior margin to weakly reticulate on most its surface, well delimited, lower margin virtually straight. Antenna with scape 0.9 × as long as eye height, reaching above level of vertex; pedicel about as long as F1; combined length of pedicel and flagellum 1.34 × breadth of head; flagellum almost filiform, all funiculars longer than broad; F1 1.83 × as long as broad, F6 1.5 × as long as broad; clava 2.6 × as long as broad.

Mesosoma 1.93 × as long as broad and 2.06 × as long as high. Mesoscutum 1.6 × as broad as long. Mesoscutellum as long as broad. Propodeum medially 0.5 × as long as mesoscutellum; median carina straight, reaching adpetiolar strip; plicae developed only posteriorly; median area smooth; nucha absent. Fore wing 2.41 × as long as broad; basal fold with complete row of setae; basal cell with scattered setae along submarginal vein, basal and cubital folds; speculum closed by a line of setae on cubital fold; M 0.83 × as long as P and 2.69 × as long as S; stigma small, uncus long, with four campaniform uncal sensilla.

Gaster lanceolate, longer than head plus mesosoma, 2.37 × as long as broad. Hypopygium in proximal quarter of gaster. Ovipositor sheaths projecting well beyond apex of metasoma, its length in dorsal view 0.75 × as long as syntergum.

**Male**. Unknown.

#### Distribution.

Cyprus.

#### Hosts.

Unknown.

#### Etymology.

Named after the ancient Greek goddess Aphrodite, which was worshiped in the type-locality, Yeroskipou (Aphrodite’s sacred gardens in Greek mythology, its name, combining “yeros” (ιερός, holy) and “kipou” (κήπος, garden)) (noun in apposition).

#### Notes.

*Janssoniella aphrodite* sp. nov. is distinctly smaller than most of the other species of the genus. In the key of Graham (1969), it differs from all other species by the following combination of characters: small body length (2 mm), propodeum medially 0.5 × as long as mesoscutellum, ovipositor sheaths projecting well beyond apex of metasoma, combined length of pedicel and flagellum 1.34 × breadth of head, presence of infumation in proximal part of basal cell. In the key of Tselikh (2020), apart from the presence of infumation in proximal part of basal cell, *J. aphrodite* runs to couplet 2. There, it differs from both species (*J. caudata* Kerrich, 1957 and *J. kawabatai* Tselikh, 2020) by the following combination of characters: basal cell of fore wing pilose apically; costal cell with one complete row of setae; F1 with one row of sensilla.

##### Unplaced to subfamily (*sensu*Burks et al. 2022)

**Genus *Hemitrichus* Thomson, 1878**

### Hemitrichus akrotiriensis

Taxon classificationAnimaliaHymenopteraPteromalidae

Koutsoukos & Mitroiu sp. nov.

24D6DD1B-2C7C-58D3-84D6-3D30EDEB00C5

https://zoobank.org/13FEEF95-E883-4DD0-9707-C46226F03D89

Fig. 5

#### Material examined.

Holotype female: Cyprus • Akrotiri U.K. Sovereign Base Area, Akrotiri village, 34.600447, 32.952179, 17.xii.2025, from beating-sheet on *Ficus benjamina* L., leg. Koutsoukos E., Demetriou J., “ZMUA000316743” (ZMUA). Paratype female: Cyprus • Paphos, Paphos, Kato Paphos, 34.776036, 32.409094, 14.xii.2025, from beating-sheet on *Ficus microcarpa* L., leg. Koutsoukos E., Demetriou J., “ZMUA000316744” (ZMUA).

**Figure 5.**
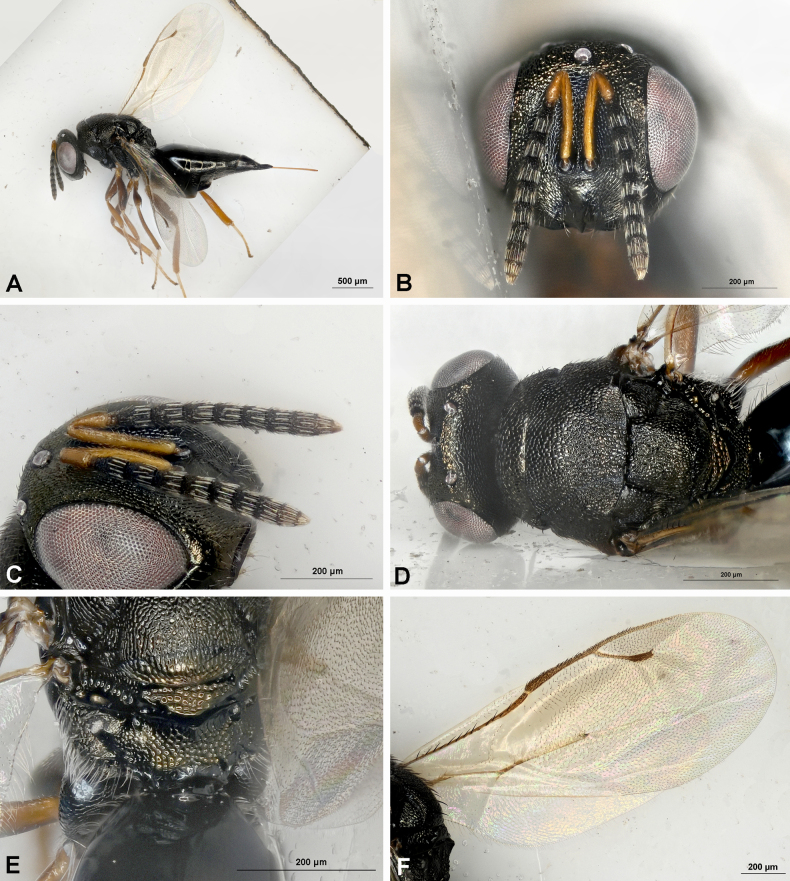
*Hemitrichus akrotiriensis* sp. nov. holotype ♀. **A**. Habitus; **B**. Head, frontal view; **C**. Left antenna, anterolateral view; **D**. Mesosoma, dorsal view; **E**. Scutellum, propodeum, dorsal view; **F**. Fore wing.

#### Description.

**Female**. Body length 2.1–2.7 mm.

Colour. Head and mesosoma black with dark-blue and dark-green metallic reflections; metasoma including ovipositor sheath black, with traces of dark-blue reflections; antenna with scape testaceous; pedicel testaceous to light brown, first anellus testaceous, rest of flagellum dark brown to blackish; all coxae as mesosoma, all femora dark brown; all tibiae mostly dark reddish brown; fore tarsus testaceous to brown, mid and hind tarsi proximally yellowish, remainder light brown; fore wing with venation and setae dark brown; setae brown or black.

Head in dorsal view 2.18–2.24 × as broad as long and 1.04–1.11 × as broad as mesoscutum; in frontal view 1.16–1.20 × as broad as high. In dorsal view temple 0.21–0.25 × as long as eye. POL 2.62–3.05 × OOL. Eye height 1.40–1.51 × eye length and 2.71–3.18 × as long as malar space. Oral fossa 0.46–0.47 × as long as head width. Clypeus smooth, its lower margin with a median tooth. Antenna 11264, inserted above LOL; scape 0.70–0.73 × as long as eye height, not reaching median ocellus or vertex; pedicel 1.09–1.57 × as long as F1; combined length of pedicel and flagellum 0.96–0.98 × breadth of head; flagellum almost filiform, all funiculars longer than broad; F1 1.40–1.83 × as long as broad, F6 1.00–1.08 × as long as broad; clava 2.23–2.66 × as long as broad.

Mesosoma 1.40–1.47 × as long as broad and 1.51–1.58 × as long as high. Pronotum 0.31–0.37 × long as mesoscutum. Mesoscutum 1.74–1.77 × as broad as long. Mesoscutellum as long as broad. Propodeum medially 0.56–0.57 × as long as mesoscutellum; median carina traceable only in upper third of propodeum and produced as a tooth; basal foveae present; plicae absent; median area mainly reticulate; nucha constricted, mainly smooth. Fore wing 2.25–2.34 × as long as broad; basal fold with complete row of setae; basal cell bare; speculum open; M 0.94 × as long as P and 1.31–1.56 × as long as S; stigma small to very elongate, its height 0.26–0.39 × as long as distance to P, uncus short.

Gaster lanceolate, 1.12–1.13 × as long as head plus mesosoma, 2.37–2.48 × as long as broad. Last tergite 1.00–1.26 × as long as its basal breath. Hypopygium reaches slightly beyond middle of gaster. Ovipositor sheaths projecting beyond apex of metasoma.

**Male**. Unknown.

#### Distribution.

Cyprus (U.K. Akrotiri Sovereign Base Area).

#### Host.

Unknown.

#### Etymology.

Named after the type locality, Akrotiri village (U.K. SBA, Cyprus) (adjective).

#### Notes.

*Hemitrichus akrotiriensis* sp. nov. differs from all other species of the genus by its larger POL:OOL ratio, the large eye length: malar space length ratio, as well as the smaller M:S ratio. More comparative characters with the other *Hemitrichus* species are presented in Table 1.

**Table 1.** Comparative characters for known Palaearctic *Hemitrichus* species.

	***H. longigaster***	***H. oxygaster***	***H. seniculus***	***H. sugonjaevi***	***H. akrotiriensis***
Gaster l:w	3.4	2.8–3.3	2.3–2.5	2.4	2.37–2.48
Gaster:thorax+head	1.94	Nearly or quite 1.5	At most slightly longer	1.2	1.12–1.13
Last tergite l:basal w	2	Nearly or quite 2	Not/hardly longer	1.3	1.00–1.12
MV:SV	2.46	1.8	1.7–1.75	1.6	1.31–1.56
Eye length:malar space	2.33	3	2.4	1.8	2.71–3.18
Fu1 l:w	Slightly longer than broad	1.6	1.3	1	1.40–1.66
Fu6 l:w	Broader than long	Hardly > 1	0.9	Broader than long	1.00–1.08
POL:OOL	2.41	2	2.2	1.6	2.62–3.10

## Discussion

The present study provides the description of five new pteromalid species from the island of Cyprus and is a small step towards enriching our knowledge of the true biodiversity and endemism of the island. With only four endemic chalcid wasp species known so far (*Dicarnosis lisenchiae* Fusu, 2012; *Proconura pseudonebulosa* (Masi, 1934); *Tetrastichus cyprus* Hansson & Schmidt, 2020; *Torymus cyprianus* Graham & Gijswijt, 1998), the description of five additional species more than doubles the number of endemic chalcid wasps of Cyprus, further highlighting the knowledge gaps and the need for further research (Masi 1934; Graham and Gijswijt 1998; Fusu 2012; Hansson and Schmidt 2020). Additionally, dozens of new records for the family Pteromalidae (and perhaps more new species) from Cyprus are to be published in a separate study.

While all the included genera have many representatives in Europe, none of their species have been detected in Cyprus. This comes to no surprise, while many pteromalid genera have been revised in the last decades (e.g. Baur et al. 2014; Tselikh 2020), the systematics and ecology of most of them remain poorly known. Based on the results above, it is evident that while Europe and its adjacent areas have been well studied regarding their pteromalid fauna (especially compared to other regions of the world), still a lot of species remain undescribed.

## Supplementary Material

XML Treatment for Ablaxia makrisi

XML Treatment for Ablaxia toxeftrae

XML Treatment for Erythromalus makrisi

XML Treatment for Janssoniella aphrodite

XML Treatment for Hemitrichus akrotiriensis

## Additional information

### Artificial Intelligence (AI) use

The authors accept full responsibility for the content of the manuscript, including the disclosure of any use of AI.

No AI tools were used in the preparation of this manuscript.

### Author ORCIDs

E. Koutsoukos https://orcid.org/0000-0002-6412-2715

M.-D. Mitroiu https://orcid.org/0000-0003-1368-7721

### Data availability

All of the data that support the findings of this study are available in the main text.
